# Circulating tumor cells prior to initial treatment is an important prognostic factor of survival in non-small cell lung cancer: a meta-analysis and system review

**DOI:** 10.1186/s12890-019-1029-x

**Published:** 2019-12-26

**Authors:** Sha-Sha Jiang, Bo Deng, Yong-Geng Feng, Kai Qian, Qun-You Tan, Ru-Wen Wang

**Affiliations:** Department of Thoracic Surgery, Institute of Surgery Research, Daping Hospital, Army Medical University, Chongqing, 400042 People’s Republic of China

**Keywords:** Circulating tumor cells, Non-small cell lung cancer, Prognosis, Meta-analysis

## Abstract

**Background:**

Our study aimed to verify the prognostic value of circulating tumor cells (CTCs) prior to initial treatment on survival of non-small cell lung cancer (NSCLC) by using meta-analysis and system review of published studies.

**Materials and methods:**

The PubMed, EMBASE and Cochrane Library were searched, respectively, to identify all studies that addressed the issues of CTCs prior to initial treatment and progression-free survival (PFS) and overall survival (OS). Finally, ten citations were included for analysis and assessment of publication bias by using review manager 5.3 statistical software and STATA 15.0.

**Results:**

Randomized model analyzing multivariate Cox Proportional Hazards Regression indicated that higher abundance of CTCs significantly predicts poorer prognosis of lung cancer cases basing both on PFS (Z = 2.31, *P* = 0.02) and OS of advanced cases (Z = 2.44, *P* = 0.01), and systematic study aslo indicated the similar results.

**Conclusion:**

High CTCs prior to initial treatment can predict shorter PFS and OS in NSCLC, and further studies are warranted in the future.

## Background

Circulating tumor cells (CTCs) are a variety of tumor cells which are detached from primary site, destroy the integrity of the base membrane, and enter the peripheral blood [1]. The cells have the ability to spread through blood and form distant metastasis in an appropriate microenvironment [2].

The underlying mechanism of how CTCs shed into the peripheral blood circulation is still unclear. Animal experiments showed that this process may be related to epithelial-mesenchymal transition (EMT), which enable epithelial cells to be highly invasive [3]. However, most of CTCs may develop apoptosis or be killed by immune system, leading to the survival of very few to form remote metastasis [4]. Thereafter, it is very critical to verify the underlying clinical significance of CTCs monitoring in cancer cases.

CTCs detection is also widely used to predict prognosis of a variety of lung tumors, including small cell lung cancer, squamous cell lung carcinoma, lung adenocarcinoma and large cell lung cancer. However, the results are largely inconclusive. Higher number of CTCs before treatment and after two cycles of chemotherapy were found to be closely related to higher stage and worse prognosis of small cell lung cancer [5]. Furthermore, CTCs and CTC clusters may be potentially predictable for risk of recurrence and short survival in early non-small cell lung cancer patients (NSCLC) [6]. However, another study showed that CTCs before treatment had no significant relationship with the overall survival of non-small cell lung cancer patients [7].

This study aimed to verify the predictive power of CTCs prior to treatment on the prognosis in non-small cell lung cancer, which may be an important disease screening and guideline of the accurate treatment.

## Materials and methods

### Data sources and searches

**Fig. 1:**
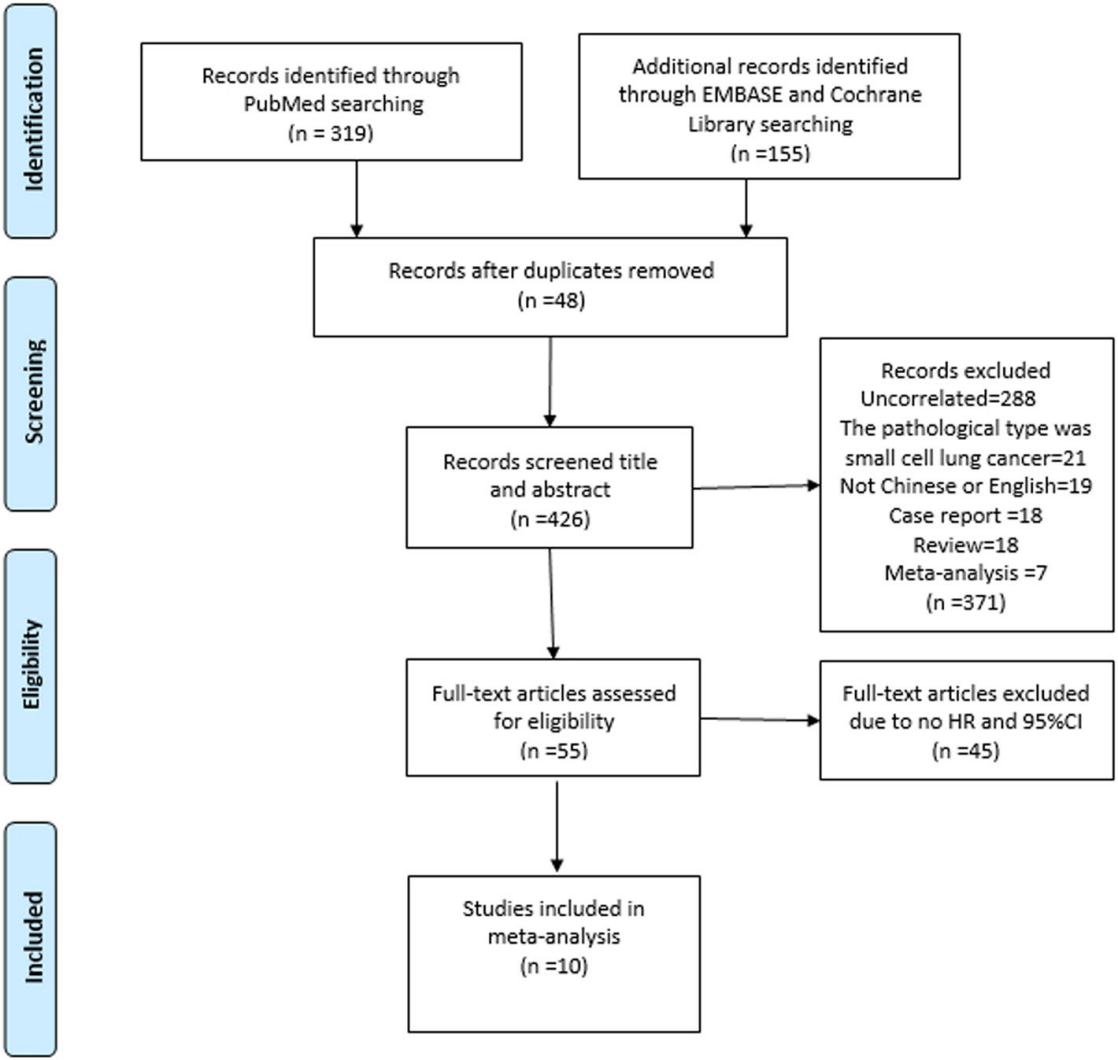
Flow chart showing the flow of publications via the review process

Medline and manual searches were performed by Jiang SS and DB, independently and jointly to identify all publications that addressed relationship between CTCs and prognosis of non-small lung cancer. Three MESH TERMs “Lung Neoplasms”, “Prognosis”and “Neoplastic Cells, Circulating” were searched in the PubMed, EMBASE and Cochrane Library database, respectively (Fig. 1).

We excluded the studies uncorrelated with NSCLC, and written in neither Chinese nor English, and case reports, reviews, and meta-analysis. Therefore, we included those studies focusing on CTCs prior to initial treatments and progression-free survival (PFS) and overall survival (OS) by using multivariate Cox Proportional Hazards Regression (multivariate COX model) with Hazard Ratio and 95%CI. Finally, a total of 10 citations were included for analysis (Tab. 1 and Fig. 1).

**Table 1:** Summary of the published studies included in the meta-analysis and system analysis

PumID	Published year	Detection method	Cases (N)	Mean age	Tumor Stage	Pathological diagnosis	Cutoff	Biomarker	Patients with high level of CTCs (n)
29,110,843	2017	size-based microfilter assay	40	67	I/II/III	Adenocarcinoma	≥1/7.5 mL	N.A.	15
28,919,743	2017	NanoVelcro	143	(−)	I/II/III/IV	Adenocarcinoma	≥1/1 mL	CK+/CD45−/DAPI+,	37
28,633,480	2017	CellSearch	125	61	I/II/III	Squamous/ Adenocarcinoma/Others	≥5/7.5 mL	CK+/CD45−/DAPI+,	24
28,492,516	2017	ScreenCell Cyto	73	67	IV	Squamous/ Adenocarcinoma	> 6/3 mL	N.A.	34
28,474,575	2017	CellSearch	107	65	III/IV	NSCLC	≥5/7.5 mL	CK+/CD45−/DAPI+,	17
28,289,866	2017	CellSearch	59	60	III/IV	Squamous/ Adenocarcinoma/Others	≥2/7.5 mL	CK+/CD45−/DAPI+,	24
27,983,527	2017	CellSearch	100	62	III/IV	Squamous Cancer	≥5/7.5 mL	CK+/CD45−/DAPI+,	9
26,661,896	2015	Cyttel method	46	(−)	III/IV	Squamous/ Adenocarcinoma	≥8/3.2 mL	CD45-	7
21,422,424	2010	CellSearch	101	67	III/IV	Squamous/Poorly differentiated/ Adenocarcinoma/Others	≥5/7.5 mL	CK+/CD45−/DAPI+,	9
21,098,695	2010	ISET	208	63	I/II/III/IV	Squamous/ Adenocarcinoma/Large cell carcinoma/Sarcomatoid carcinoma	≥50/10 mL	N.A.	64

Note:

N.A: Not addressed

Levels of evidence were evaluated according to the recommendation of the American Academy of Orthopedic Surgeons Evidence-Based Practice Committee, and a trial quality score was assessed according to the method of Jadad et al. [8]. The evaluation of CTCs as a prognostic factor was performed by analyzing the hazard ratio (HR) and their 95% confidence interval (CI), using randomized model, due to heterogeneity among the studies. The publication bias of selected studies was assessed by means of Egger’s regression test and funnel plot, and *P* < 0.05 was considered the existence of publication bias. Meanwhile, Green I statistics were used to assess heterogeneity. Stratified analysis was used to identify the potential confounders leading to heterogeneity. I^2^ > 50% indicated the existence of significant heterogeneity. All statistical analysis was calculated by using Review Manager 5.3 for meta-analysis and STATA 15.0 for evaluation of publication bias, respectively.

## Results

**Fig. 2:**
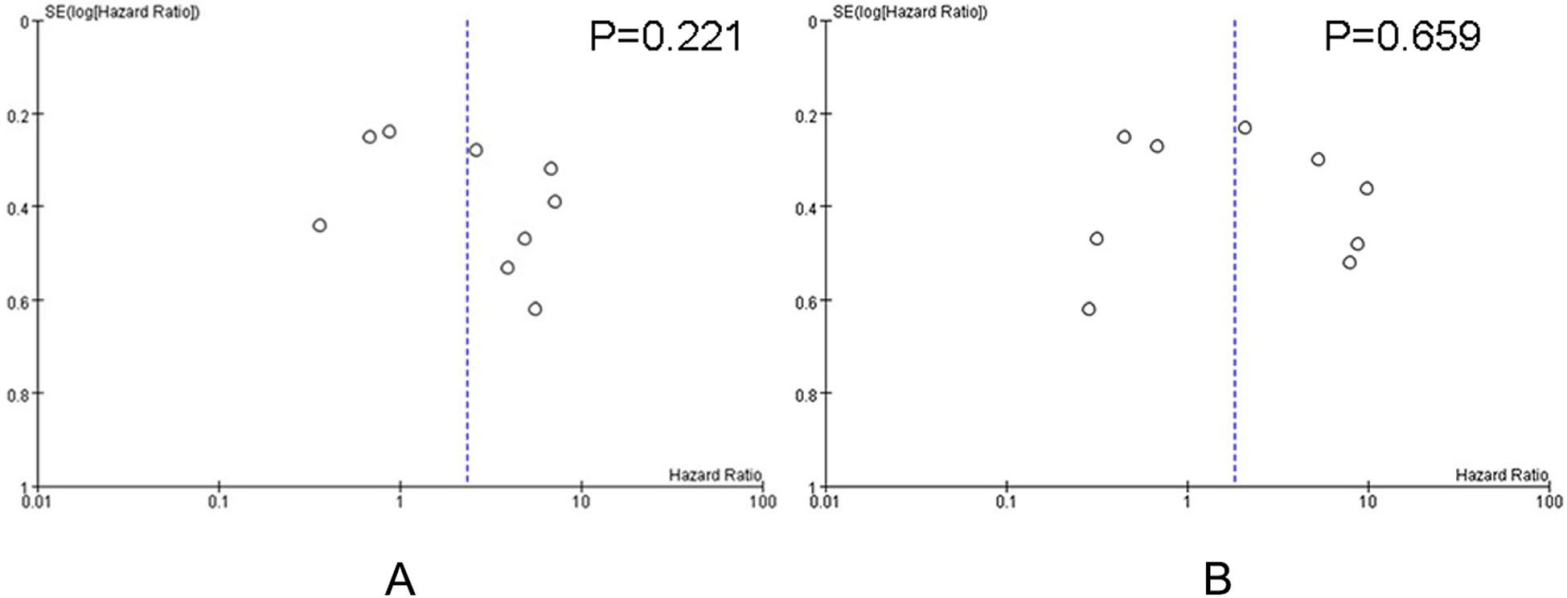
Funnel plot verifies publication bias of studies. It unveiled that most studies are at the top of the inverted funnel, while there are few studies at the base, and the left and right are roughly symmetrical, so the publication bias is not obvious. **a** Funnel plot of studies focusing on PFS,*p* = 0.221. **b** Funnel plot of studies focusing on OS, *p* = 0.659

Publication bias test regarding PFS and OS unveiled most studies were at the top of the inverted funnel, while very few studies were at the base, and the left and right are roughly symmetrical. Additionally, Egger’s regression test result showed that the *P* values of PFS and OS were 0.221 and 0.659 respectively,indicating the publication bias was not remarkbale (Fig. 2a and b).

### Higher abundance of CTCs predicts shorter PFS of lung cancer cases

**Fig. 3:**
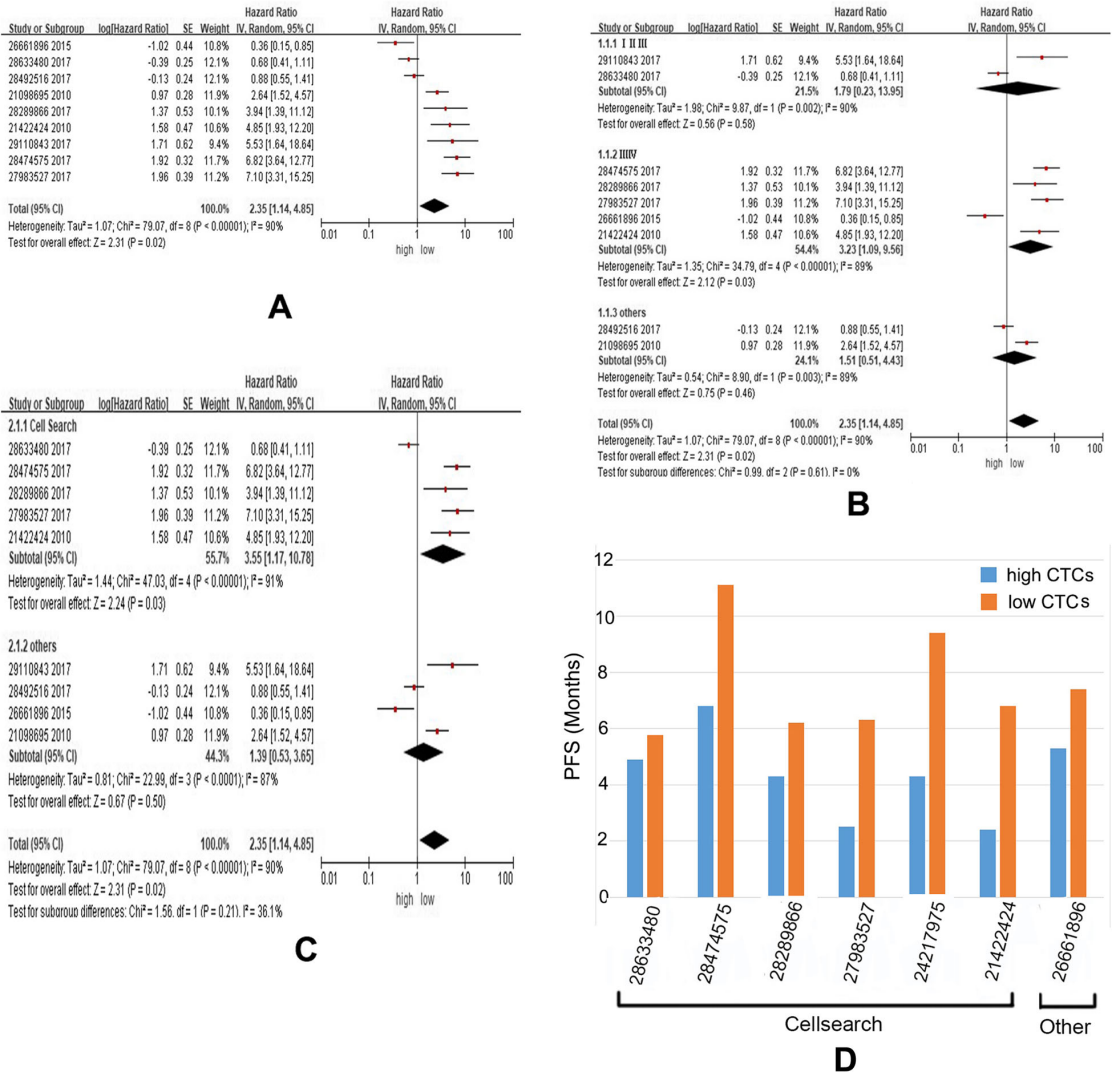
Evaluation of PFS with high or low abundance of CTCs by using meta-analysis (**a**-**c**) and systematic review (**d**). **a** Higher abundance of CTCs predicts shorter PFS of NSCLC. **b** Stratified study found that the differences of heterogeneity among tumor stages are quite small(I^2^ = 0%). **c** Stratified study found that the differences of heterogeneity among various detect methods of CTCs are moderate(I^2^ = 36.1%). **d** Systematic review of PFS (months) data indicated higher abundance of CTCs are asscociated with shorter PFS

Randomized model analyzing multivariate Cox Proportional Hazards Regression indicated that higher abundance of CTCs significantly predicts shorter PFS of lung cancer cases (Z = 2.31, *P* = 0.02), however, heterogeneity among the studies was also considered to be remarkable(χ^2^ = 79.07, *P* < 0.00001; I^2^ = 90%) (Fig. 3a). Further stratified studies indicated the differences of heterogeneity among tumor stages were quite small (I^2^ = 0%) (Fig. 3b). Intriguingly, the abovementioned differences among various methods to detect CTCs were moderate (I^2^ = 36.1%) (Fig. 3c), potentially leading to the abovementioned heterogeneity in Fig. 3a.

Systematic study of current available data of PFS (months) also indicated higher abundance of CTCs appeared in the cases with shorter PFS (Fig. 3d).

### Higher abundance of CTCs predicts shorter OS of advanced lung cancer cases

**Fig. 4:**
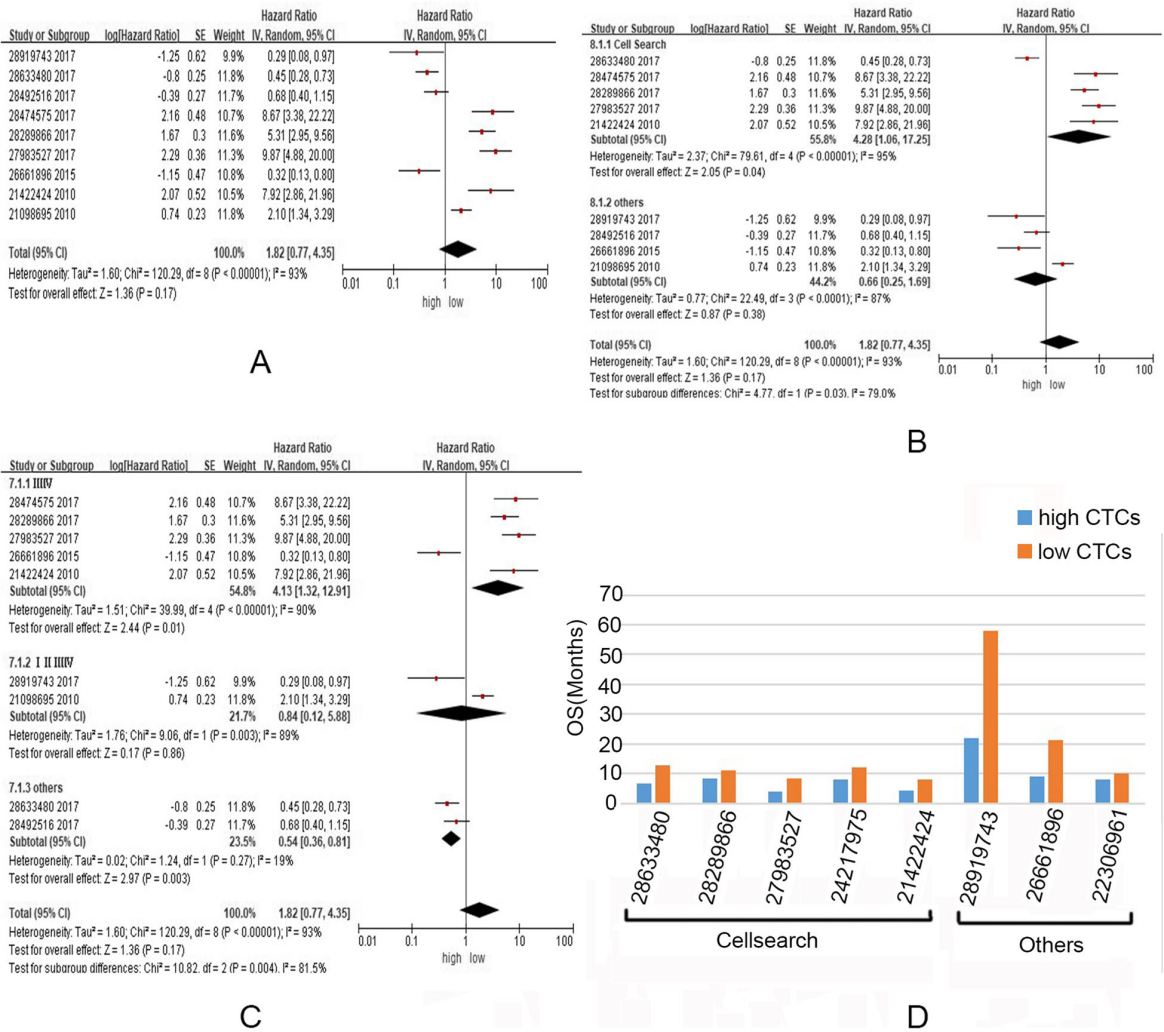
Evaluation of OS with high or low abundance of CTCs by using meta-analysis (**a**-**c**) and systematic review (**d**). **a**. Higher abundance of CTCs had not significant predict power of prognosis of lung cancer cases. **b** Stratified analysis showed the differences of tumor stages in different cohorts lead to heterogeneity(I^2^ = 81.5%). **c** Stratified analysis showed various detect methods in different cohorts lead to heterogeneity (I^2^ = 79.0%). **d** Systematic review of OS (months) data indicated higher abundance of CTCs are associated with shorter OS

Randomized model analyzing multivariate Cox Proportional Hazards Regression indicated that abundance of CTCs had no significant predictive power in total cohort mixing later and early stages (Z = 1.36,*P* = 0.17),however, heterogeneity test suggested significant heterogeneity among studies (χ^2^ = 120.29, *P* < 0.00001; I2 = 93%) (Fig. 4a). Stratified analysis showed the differences of tumor stages in different cohorts lead to the abovementioned heterogeneity (I^2^ = 81.5%), and higher abundance of CTCs seemed to had significant predict power in advanced cases (Z = 2.44, *P* = 0.01) (Fig. 4b). Intriguingly, various detect methods also could result in heterogeneity (I^2^ = 79.0%). Indeed, we focused on the only studies by using CellSearch, and found the CTCs had more significant predicted power (Fig. 4c).

Systematic study of current data of OS (months) also indicated higher abundance of CTCs appeared in the cases with shorter OS (Fig. 4d).

## Discussion

The appearance of CTCs in peripheral blood is often thought to be critical to remote tumor metastasis. Therefore, CTCs detection is considered to be an effective and non-invasive method that can be applied to diagnosis, and evaluation of treatment response, prognosis and recurrence risk of a variety of malignant tumors [9–11]. Thus far, various methods, e.g., CellSearch, High-gradient magnetic cell sorting (MACS), isolation by size of epithelial tumor cells (ISET), RT-PCR, Ficoll, OncoQuick and CTC-chip for detecting CTCs has been introduced. The abovementioned technologies for CTCs detection can be divided into EpCAM-based, e.g., CellSearch, MACS and CTC-chip or EpCAM-independent methods, e.g., ISET, RT-PCR, Ficoll and OncoQuick respectively.

CellSearch platform was the only method approved by the United States food and drug Administration (FDA) and commercially used for CTCs detection, which was designed to enrich CTCs basing on epithelial cell adhesion molecule (EpCAM). EpCAM-positive cells are enriched by immunomagnetic separation using EpCAM-specific antibodies conjugated to magnetic particles and then stained with fluorescent anti-cytokeratin and 40,6diamino-2-phenylindole (DAPI), while hematopoietic cells are stained with anti-CD45 antibodies [12]. Thereafter, Semiautomated fluorescent microscope can be used to verify and count Cytokeratin(+)-DAPI (+)-CD45(−) CTCs [13]. Whilst EpCAM is expressed in the majority of CTCs, EMT can downregulate epithelial biomarkers including EpCAM, leading to the miss of a subpopulation of CTCs during enrichment or detection [14]. Furthermore, the specificity of CellSearch was also proved to be poor in normal individuals and in patients with benign tumors [15, 16].

MACS was designed to magnetically specifically label CTCs with an EpCAM, anti-cytokeratin 8 monoclonal antibody (mAb) which was directly conjugated to the superparamagnetic microbeads [17]. Magnetic beads targeting the tumor-specific cell antigen human epidermal growth factor receptor 2 (HER2) are also available [18]. Thereafter, CTCs are detected in the enriched cell fraction by flow cytometry, fluorescence microscopy, or immunocytochemistry [19]. This technique can highly deplete leukocytes and guarantee an efficient enrichment of tumor cells. As Cell search, EpCAM (−) CTCs cannot be detected by MACS.

ISET allows the counting and immunomorphological and molecular characterization of CTCs which are isolated and collected by filtration via an 8 μm-pores filtering membrane, due to larger size of tumor cells as compared with peripheral blood leukocytes [20]. Enriched cells are stained on the filter for cytomorphological, immunocytochemical or further examination [21]. While this method is convenient and economic, it lacks specificity because smaller tumor cells may be filtrated and ignored, or larger blood cells may be mistakenly collected and determined as tumor cells [22].

Ficoll density gradient centrifugation method is also based on cell morphology. In density centrifugation systems, erythrocytes, platelets, and polymorph nuclear cells are separated in the pellet, while mononuclear cells (MNCs), including CTCs, are collected in the so-called interphase which can be used for the further detection of tumor cells by immunocytochemistry or RT-PCR [23]. This method is relatively convenient and economic for all tumor types, but has poor specificity and low purification of isolated cells [9]. OncoQuick system, using multi-empty partitions to enrich tumor cells, was developed to satisfactorily reduce the co-enriched MNCs, with a high tumor cell recovery rate, and increase the chance of detecting CTCs [24].

Detection of mRNA that are overexpressed or mutated in cancer using RT-PCR is a more widely used as alternative. As RNA disappears quickly after cell death, detection of RNA is likely due to the presence of a whole tumor cell, not cell fragments or free RNA [25]. Theoretically, the sensitivity of RT-PCR is higher than immunocytochemistry, and false-positivity of RT-PCR is relatively high due to sample contamination, expression of target genes in normal cells, and pseudo genes. Collectively, RT-PCR with it’s higher sensitivity and lower specificity, as background noise due to expression of markers in normal cells, is hard to distinguish from a true positive signal [26]. Expectedly, optimization of combination of multi-markers assay may increase the accuracy of RT-PCR as adjuvant diagnosis method of lung cancer [27].

Recently, a novel method, i.e., CTC-chip is developed to detect CTCs [28] This chip consists of 78,000 micro posts coating with EpCAM antibodies and binds EpCAM-positive cells to the microposts, while whole blood is pumped through the system. Thereafter, a camera is used to detect the trapped cells basing on their morphology, viability and other tumor markers, e.g., CKs (+) and DAPI (+) and CD45(−). CTC-chip can result in specific enrichment and visual confirmation of CTCs, but may miss EpCAM negative CTCs [29].

Totally, the sensitivities of EpCAM-based detection methods seemed to be significantly lower than EpCAM-independent detection methods [30] [31] [32] due to the down-regulation of EpCAM in cancer cells during epithelial-mesenchymal EMT process. Therefore, CanPatrol™ is currently developed to classify and detect CTCs, as per both EMT markers, i.e., epithelial biomarkers (EpCAM and cytokeratins) and mesenchymal biomarkers (vimentin and twist) [33]. Therefore, CTCs can be clustered into three subtypes, i.e., CTCs with epithelial markers, CTCs with mesenchymal markers, and CTCs with both. Pilot study unveiled that detection of CTCs by using CanPatrol™ can effectively monitor tumor progression after operation in lung cancer cases [34]. Classifying CTCs by EMT markers helps to identify the more aggressive CTCs subpopulation. However, the discrimination and distinguishing of those biomarkers in CTCs as molecular prognostic factors warrants further robust study [35].

Detection of CTCs has been widely used for prediction of prognosis in a variety of malignancies, e.g., prostate cancer, breast cancer, neuroendocrine cancer and colorectal cancer. In patients with new metastatic hormone-sensitive prostate cancer, lower baseline CTCs (prior to treatment) were associated with higher rate of prostate-specific antigen (PSA) response [36]. The similar result was verified by another study on metastatic castration-resistant (which cannot be treated by excision) prostate cancer [37]. Another study on breast cancer confirmed the independent prognostic effect of CTC count on PFS and OS that higher CTC count prior to treatment was associated with decreased PFS and OS compared patients with lower CTC count [38]. Similarly, the presence of CTCs was associated with worse PFS and OS in neuroendocrine cancer patients [39]. Meanwhile, in patients with stage I and II colorectal cancer. CTC status was the only variable significantly associated with cancer relapse [40].

For meta-analysis, we only incorporated the data of multivariate, rather than univariate, Cox-regression model because multivariate model can adjust the influence of other prognostic factors, e.g., tumor stage and age, hence, it is more reliable. Our study revealed higher count of CTCs was associated with shorter PFS and OS of NSCLC. Furthermore, CTCs has higher predictive power for PFS as compared with OS, probably due to other demographical and clinical confounders, e.g., age, physical conditions, and compliance to the subsequent treatment after recurrence, which may influence OS. Intriguingly, stratified meta-analysis indicated that the predictive power of OS is more significant in stage III/IV patients than in other stage.

The heterogeneity among studies was significant(I^2^ ≥ 50%), probably due to the differences of detection methods, cut off values, and pathological stages used in different studies. We tried to reduce the heterogeneity by stratifying the studies into subgroups according to the pathological stage and detection method in meta-analysis. Intriguingly, the heterogeneity was decreased remarkably, however, it still cannot be eliminated totally. For instance, the different cut-off values were used by the studies which used CellSearch which was approved by FDA for breast cancer without recommended cut-off value for lung cancer. Therefore, the studies took the different cut-off values referring to breast cancer or their own pilot study results. Additionally, we attempted to decrease the publication bias by searching studies completely, although no significant publication bias was found in the result of Egger’s regression test. Inevitably, some drawer data will be missed.

## Conclusion

High CTCs prior to initial treatment can predict shorter PFS and OS in NSCLC. It may provide an important evidence for precision and personalized therapy in lung cancer, for instance, the cases with higher level of CTCs prior to initial treatment or without satisfactory decrease trend of CTCs following treatment are supposed to undergo more fierce treatment. Furthermore, the efficient detection methods of CTCs with high sensitivity and specificity are required to be explored, and further robust clinical studies are warranted focusing on CTCs as prognostic factor of NSCLC patients.

## Data Availability

The dataset was searched on PubMed (http://www.ncbi.nlm.nih.gov), EMBASE (https://www.embase.com/), Cochrane Library (https://www.cochranelibrary.com/). The search strategies and yielded citations were shown in Tables 1 and Fig. 1, respectively.

## Abbreviations

CTC: Circulating tumor cell
NSCLC: Non-small cell lung cancer
